# Preclinical Evaluation of Stable Integrin α_v_β_3_-Specific [^198^Au]Gold Nanoparticles for Tumor Therapy

**DOI:** 10.3390/ph18111670

**Published:** 2025-11-04

**Authors:** Güllü Davarci, Carmen Wängler, Klaus Eberhardt, Margaret Tulessin, Christopher Geppert, Ralf Schirrmacher, Gert Fricker, Carolin Mogler, Marc Pretze, Björn Wängler

**Affiliations:** 1 Molecular Imaging and Radiochemistry, Clinic of Radiology and Nuclear Medicine, Medical Faculty Mannheim of Heidelberg University, 68167 Mannheim, Germany; guellue.davarci@medma.uni-heidelberg.de; 2 Research Campus M^2^OLIE, Medical Faculty Mannheim of Heidelberg University, 68167 Mannheim, Germany; 3 Biomedical Chemistry, Clinic of Radiology and Nuclear Medicine, Medical Faculty Mannheim of Heidelberg University, 68167 Mannheim, Germany; carmen.waengler@medma.uni-heidelberg.de; 4 Forschungsreaktor TRIGA Mainz, Johannes-Gutenberg-Universität Mainz, 55128 Mainz, Germany; eberha@uni-mainz.de (K.E.); cgeppert@uni-mainz.de (C.G.); 5 Institute of Pathology, School of Medicine and Health, Technical University of Munich (TUM), 81675 Munich, Germany; margaret.tulessin@tum.de (M.T.); carolin.mogler@tum.de (C.M.); 6 Division of Oncological Imaging, Department of Oncology, University of Alberta, Edmonton, AB T6G 2R3, Canada; schirrma@ualberta.ca; 7 Institute of Pharmacy and Molecular Biotechnology, Heidelberg University, 69120 Heidelberg, Germany; gert.fricker@uni-hd.de; 8 Department of Nuclear Medicine, University Hospital Carl Gustav Carus, TU Dresden, 01307 Dresden, Germany; 9 Mannheim Institute for Intelligent Systems in Medicine MIISM, Medical Faculty Mannheim of Heidelberg University, 68167 Mannheim, Germany

**Keywords:** gold nanoparticles, [^198^Au]AuNPs, radioactive, tumor therapy, tumor angiogenesis, RGD peptide

## Abstract

**Objectives**: This paper reports the preclinical evaluation of stable tumor-specific gold nanoparticles (AuNPs) activated by neutron irradiation as a therapeutic option for the treatment of cancers characterized by high tumor angiogenesis. **Methods**: A selection of promising AuNPs with high avidity to α_v_β_3_-expressing glioma (U-87 MG) cells (IC_50_ = 82–104 nM) were chosen with different surface loading of Arg-Gly-Asp (RGD) peptides as tumor targeting vectors for integrin α_v_β_3_, a target which is overexpressed in tissues displaying high tumor angiogenesis. Three different [^198^Au]AuNPs were evaluated applying three injection methods, intravenous (i.v.), intraperitoneal (i.p.), and intratumoral (i.t.), each in a group of six U-87 MG xenograft–bearing mice (54 female athymic nude mice in total). Their biodistribution and tumor accumulation was assessed by in vivo imaging within 1–7 days after injection and 7 days after injection by ex vivo measurement. **Results**: The developed [^198^Au]AuNPs exhibited suboptimal biodistribution by i.v. application (accumulation pattern tail > liver > spleen, no significant tumor accumulation) and by i.p. application (accumulation pattern spleen >> liver > pancreas, slight tumor accumulation of <0.3 %ID/g). However, an acceptable biodistribution by i.t. application was observed (5.5 %ID/g in liver, 4.9 %ID/g in spleen, and 3.0 %ID/g in tumor). **Conclusions**: Despite the very promising in vitro results, the in vivo evaluation suggests that the [^198^Au]AuNPs represent a platform for the development of restricted therapeutic strategies.

## 1. Introduction

^198^Au-labeled, radioactive gold nanoparticles ([^198^Au]AuNPs) are promising compounds for application in nanobrachytherapy of malignant diseases, as they exhibit no metal-related toxicity, unlike other particulate metal-based compounds. First described in the 1950s for this purpose [1,2], these nanoparticles have garnered significant attention due to their potential benefits. Notably, very small nanoparticles with a size of <5 nm [3] are particularly relevant in this regard, as they exhibit renal excretion and rapid clearance. The particles can be taken up into the tumors through an unspecific effect known as the “enhanced permeability and retention (EPR) effect” [4], which is attributed to the unusually large fenestration of endothelial cells of tumor vessels. Furthermore, gold nanoparticles offer the possibility of functionalizing their surface with tumor-specific compounds, thereby increasing their tumor targeting ability [5,6].

As gold nanoparticles have been identified as promising carriers for tumor imaging and therapy [7,8,9,10,11,12,13], various approaches have been developed in recent years to enhance their bioavailability, optimize renal excretion [14], and modify their surface properties. These efforts have included PEGylation to improve bioavailability [15,16], optimization of particle size for enhanced renal clearance, and the use of small molecules [17], peptides [18,19], radionuclides [20,21,22,23,24], or near-infrared dyes [25,26] for surface modification.

Stable and efficient surface modification of gold nanoparticles can be achieved by conjugating thiol-functionalized molecules [27], which exhibit high affinity towards the gold nanoparticle surface [28] forming Au-S bonds. Recently, it has further been demonstrated that bidentate dithiol compounds enable even more stable conjugation to the particles [29].

For the therapeutic application in tumor therapy, [^198^Au]AuNPs are of special interest as they can be used as radiosensitizers by Auger–Meitner electron emission induced by gamma activation [30,31,32] or by direct neutron activation of natural ^197^AuNP, generating [^198^Au]AuNPs (t_1/2_ = 2.69 d, β^−^_max_ 961 keV, 98.99%; γ 412 keV, 95.62%) [18,33,34,35,36].

The focus of the present work was the preclinical evaluation of selected highly stable targeted AuNPs for neutron activation [37] in terms of in vivo pharmacokinetics. Previous work showed that AuNPs functionalized with dithiol linkers exhibit markedly higher stability during and after neutron irradiation than their monothiol-linked counterparts [34]. Consequently, the dithiol-linked AuNPs were selected for in vivo evaluation in the present study. These particles were further functionalized with the peptidic α_v_β_3_ integrin-specific binder c(RGDfK) in order to achieve a target-specific accumulation in malignant tissues exhibiting a high fraction of angiogenesis [38]. Radiopharmaceuticals incorporating multimeric RGD motifs display markedly improved target accumulation relative to monomeric RGD analogues [39]. The RGD peptide motif exhibits high affinity for transmembrane integrin α_v_β_3_ [37,38], a receptor upregulated in the angiogenic vasculature of diverse tumors, including U-87 MG glioblastoma cells [39,40,41]. The dithiol-peptide-modified AuNPs were decorated with low or high loadings of target-specific RGD peptides to compare their biodistribution and therapeutic impact on animal xenografts. The primary objective was to identify the optimal administration route for [^198^Au]AuNPs—intravenous (i.v.), intraperitoneal (i.p.), or intratumoral (i.t.)—for their optimal therapeutic application.

## 2. Results

### 2.1. Synthesis of AuNPs and Functionalization with the Integrin α_v_β_3_-Specific Peptide c(RGDfK)

The integrin α_v_β_3_ is a transmembrane protein which is overexpressed on endothelial cells during neoangiogenesis, for example, during metastasis and tumor growth, and thus represents a potent tumor target for radioactive AuNPs decorated with RGD peptides [40]. A specific binder to this integrin is the triple amino acid sequence Arg-Gly-Asp (RGD).

**Figure 1:**
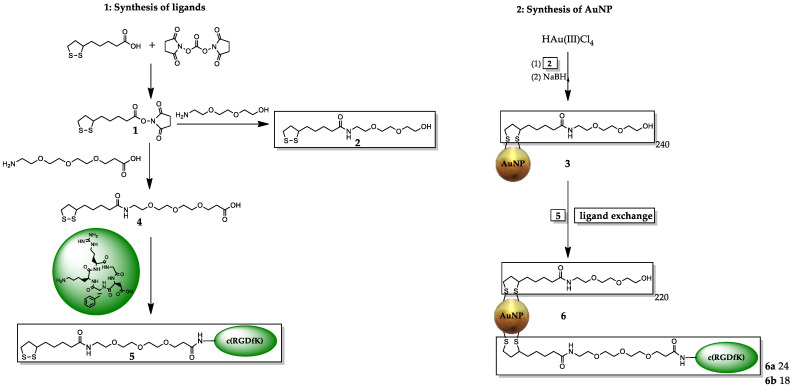
Synthesis of different RGD-functionalized AuNPs **3** (without RGD), **6a** (higher loading, 24× RGD) and **6b** (lower loading, 18× RGD).

Thus, in this work, c(RGDfK)-coated nanoparticles were intended to be synthesized, radiolabeled, characterized, and preclinically evaluated in terms of their suitability for tumor theranostics. For this purpose, ultra-small AuNPs with diameters of 3–6 nm were synthesized according to established protocols [41,42]. To enhance biocompatibility and stability of the systems, a thioctic acid (TA)-PEG_3_-OH derivative (**2**) was employed as the stabilizing ligand (Figure 1). The resulting AuNPs were further functionalized by ligand exchange with varying amounts (4–8 mg, 4–8 µmol) of a TA-PEG_4_-c(RGDfK) derivative (**5**) to obtain AuNP-dithio-RGD conjugates with high (**6a**) and low (**6b**) RGD loading, respectively (Figure 1). Purification of the functionalized Au-NPs was achieved by dialysis and size-exclusion chromatography, and the size and stability of the resulting AuNPs were confirmed by UV/Vis spectroscopy and HPLC analysis. Further, the avidity of the particles **6a** and **6b** to U-87 MG cells was evaluated using a competitive displacement assay, giving IC_50_ values of 82.4 ± 9.2 nM and 103.6 ± 3.5 nM, respectively. In contrast, the monomeric reference ligand c(RGDfK) exhibited an IC_50_ value of 700.4 ± 155.9 nM under identical conditions, highlighting the significantly enhanced cell-binding potency of the multivalent constructs.

**Figure A1:**
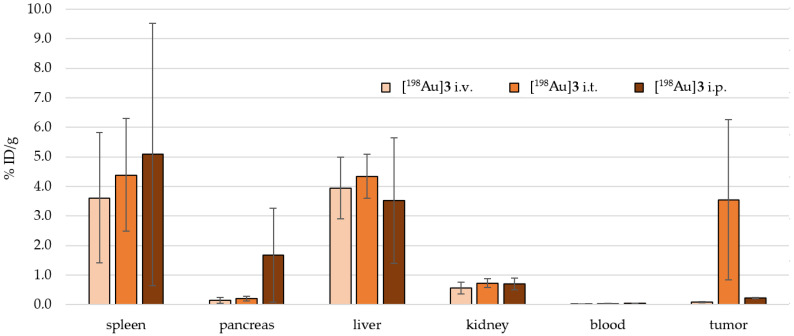
Ex vivo biodistribution of the organs of interest for different injection routes of [^198^Au]**3**.

In order to characterize the organic coating of the AuNPs, thermogravimetric analyses were performed after each functionalization step which enables the calculation of the mean number of newly attached molecules (PEG, RGD) per particle using a formula of Zhu et al. [43] (Table 1). A brief description of the synthesis and characterizations can be found in the Appendix A. All AuNPs used in this work were fully characterized by thermogravimetric analysis (TGA), electron microscopy (EM) (Figure A1), UV/Vis, HPLC, and NMR [44].

### 2.2. Neutron Irradiation of Developed AuNPs ***3***, ***6a*** and ***6b***

For theranostic purposes, the developed AuNPs were activated by neutron irradiation to produce stable [^198^Au]AuNPs. Routine neutron irradiation experiments with thermal neutrons at a TRIGA reactor located at Johannes Gutenberg University (Mainz, Germany) were performed according to a previously established protocol [44]. In brief, varying masses of AuNPs **3** and AuNPs **6a**,**b** were irradiated alongside a gold-standard reference to determine activity yields. Stock suspensions were prepared by dispersing 7–15.3 mg of AuNPs in 2 mL of 10% (*v*/*v*) ethanol containing 25 mg of ascorbic acid as a radiolytic stabilizer. The resulting activity yields are summarized in Table 2. The samples were frozen and removed from the freezer immediately before irradiation. Irradiation of samples was performed at 100 kW for 1–2 h with a neutron flux of 1.6 × 10^12^ n/cm^−2^·s^−1^. No precipitation or other form of aggregation of the activated AuNPs was observed in any sample. The activated samples were stored in the freezer for transport and further experiments.

During the irradiation of the AuNPs (**3**, **6a**, **6b**), short-lived nuclides (^24^Na, ^38^Cl, ^35^S, ^41^Ar) were also produced, and these were still measured approximately 2–5 h after irradiation. The higher activity level of the samples and their deviation from the calculated value (20–40%) are due to the short-lived nuclides identified by gamma spectroscopy (see Table A1 for details). Furthermore, sulfur is present in the samples. Its activation product (^35^S) is a pure β-emitter and not detectable by gamma spectroscopy. However, ^35^S contributes to the obtained activity as shown in Table 2. With the high-purity gold wire, the activity was measured after 26 h. The discrepancy of 10% (60 instead 66 MBq) might originate from the calibration of the ISOMED 2010 with ^137^Cs (85% 662 keV) vs. ^198^Au (96% 412 keV).

### 2.3. Cell Experiments

#### Determination of Cell Survival

Colony formation assays were performed with [^198^Au]**3** and [^198^Au]**6a**,**b** on α_v_β_3_-positive U-87 MG cells. For this proof-of-concept experiment, 5–10 Gy was chosen as the incubation dose for 300k–500k cells. To achieve this dose, the activity of 1–2 MBq [^198^Au]AuNPs per well in a 24-well plate within a 96 h incubation period was calculated using Formula (1).

(1) $$D(A,t)=S*\frac{A*T_{1/2}}{\ln(2)}\left(1-\exp\left(-\ln(2)\right)*\frac{t}{T_{1/2}}\right)$$

Calculation of the dose exerted to a cell monolayer at the bottom of a multi-well-plate or Eppendorf tube by ^198^Au using Geant4-simulation [45]. *D*: energy dose, *S*: S-value, *A*: activity, *T*_1/2_: half-life of the radionuclide, *t*: irradiation time.

**Figure 2:**
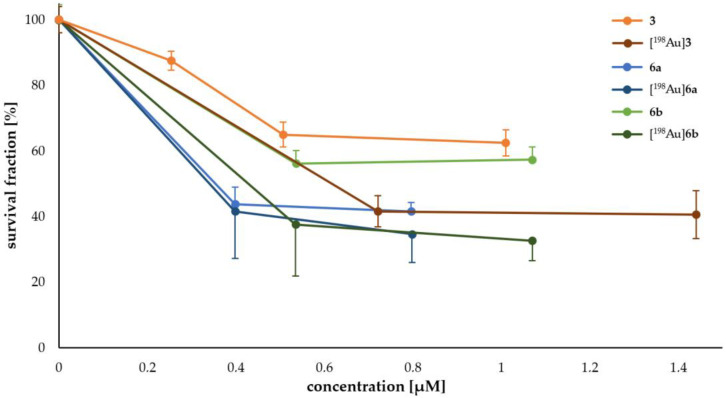
Survival fractions of the colony formation assays for different concentrations of **3** (orange), **6a** (blue), **6b** (green), [^198^Au]**3** (brown), [^198^Au]**6a** (dark blue), and [^198^Au]**6b** (dark green). Due to the production of ^198^Au, the concentration of the [^198^Au]AuNPs varies for 5 Gy and 10 Gy, and the pH was maintained at 7. At an activity curve (brown, dark blue, or dark green), the lower concentration correlates with 5 Gy, and the higher concentration correlates with 10 Gy.

The administration of 1–2 MBq of [^198^Au]AuNPs corresponded to nanoparticle concentrations of 0.40–1.44 µM, exceeding the IC_50_ values of the dithiol-RGD conjugates **6a** and **6b** by at least one order of magnitude. In clonogenic assays, treatment with [^198^Au]AuNP **3** produced a significant reduction in cell survival. Increasing the absorbed dose from 5 Gy (sf = 41.6 ± 4.8%) to 10 Gy (sf = 40.6 ± 7.3%) did not further decrease the surviving fraction (Figure 2, Table A2).

Because ascorbic acid (Asc) was used as a stabilizer during neutron irradiation, subsequent experiments led to a substantial carry over of Asc into the cell cultures. Additionally, cyclic RGD peptides are known to inhibit α_v_β_3_, which cause apoptosis of angiogenic endothelial cells, influencing the cell survival. Therefore, non-radioactive RGD-AuNPs supplemented with the equivalent concentration of Asc were evaluated prior to neutron irradiation to assess the influence of Asc—and the resulting acidification of the culture medium—on the surviving fraction (Table 3) [46,47].

At 14.2 mM Asc combined with ~2.0 µM AuNPs **3**, cell viability was completely abolished. Consequently, evaluation of RGD- and ^198^Au-mediated effects on survival was confined to Asc concentrations below 7.1 mM (~1.4 µM AuNPs-**3**). For comparison, at a concentration of 14.2 mM Asc without AuNPs, a survival fraction of 55.0 ± 9.1% was observed due to the low pH within the incubation cell media.

### 2.4. Preclinical Experiments

**Figure 3:**
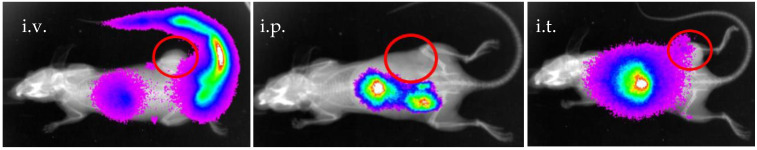
Representative in vivo planar imaging of i.v., i.p., and i.t. injections of [^198^Au]**6b** on day 3 p.i. measured with an In-Vivo Xtreme system equipped with a radiographic phosphor screen. Red circles indicate tumor localization and colors relate to activity concentrations, increasing from violet over dark blue, light blue, green, yellow, and red to white.

Animal xenografts of U-87 MG (54) were treated with 3–5 MBq [^198^Au]**3**, [^198^Au]**6a** and [^198^Au]**6b**. For every substance, three different injection routes (i.v., i.p., i.t.) were evaluated. Every injection route was tested at six animal xenografts for statistical certainty of the experiment. Therefore, 54 animals were used throughout the preclinical evaluation. After injection of the [^198^Au]AuNPs, their distribution was measured by scintillation imaging using a radiographic phosphor screen under the animals (Figure 3) at 1, 3, and 7 days post-injection (p.i.). The imaging revealed no significant differences between the different [^198^Au]AuNP species but a significant difference for the injection pathway.

**Figure 4:**
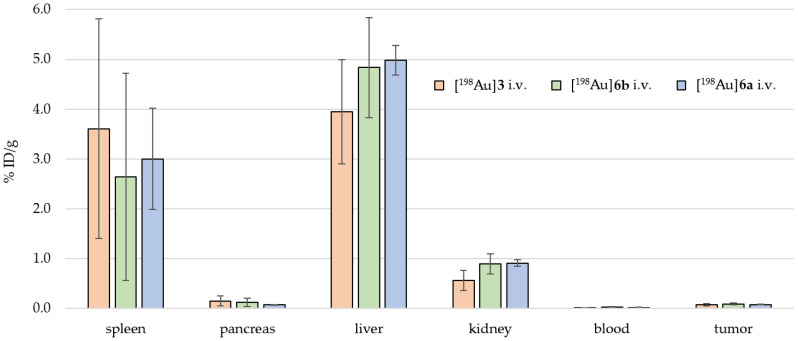
Ex vivo biodistributions of [^198^Au]**3** (orange), [^198^Au]**6a** (blue), and [^198^Au]**6b** (green) for i.v. application. Each column stands for the mean value in %ID/g per organ ± standard deviation (n = 6).

On day 7, the animals were sacrificed, and the activity of [^198^Au]AuNPs in the organs was measured by gamma-counting (Table A3). Ex vivo biodistribution of the animals revealed the following activity values for selected organs at risk (OARs) and tumor:

Figure 4 shows the biodistribution for different [^198^Au]AuNPs found for i.v. application. For [^198^Au]**3**, the highest accumulation was found in the tail (4.98 ± 2.55 %ID/g), followed by the liver (3.95 ± 1.05 %ID/g) and the spleen (3.61 ± 2.21 %ID/g), with no significant accumulation in the tumor.

For [^198^Au]**6a**, the highest accumulation was found in the tail (7.50 ± 1.03 %ID/g), followed by the liver (4.99 ± 0.29 %ID/g) and the spleen (3.00 ± 1.02 %ID/g), with no significant accumulation in the tumor.

For [^198^Au]**6b**, the highest accumulation was found in the tail (7.77 ± 3.74 %ID/g), followed by the liver (4.84 ± 1.01 %ID/g) and the spleen (2.64 ± 2.08 %ID/g), with no significant accumulation in the tumor.

**Figure 5:**
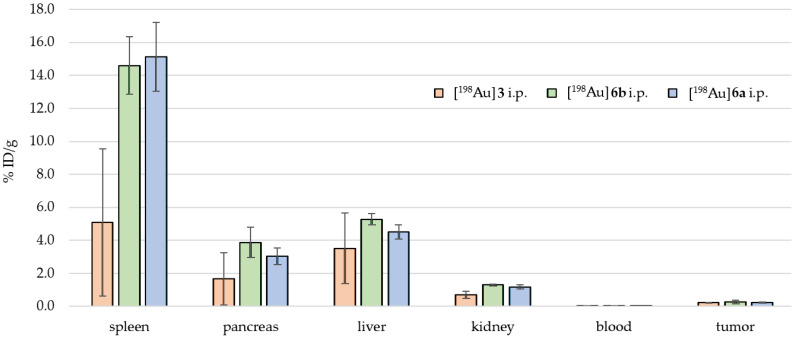
Ex vivo biodistributions of [^198^Au]**3** (orange), [^198^Au]**6a** (blue), and [^198^Au]**6b** (green) for i.p. application.

Figure 5 shows the biodistribution for different [^198^Au]AuNPs found for i.p. application. For [^198^Au]**3**, the highest accumulation was found in the spleen (5.09 ± 4.45 %ID/g), followed by the liver (3.52 ± 2.12 %ID/g), the pancreas (1.67 ± 1.59 %ID/g), and a small fraction in the tumor (0.22 ± 0.10 %ID/g).

For [^198^Au]**6a**, the highest accumulation was found in the spleen (15.12 ± 2.10 %ID/g), followed by the liver (4.51 ± 0.43 %ID/g), the pancreas (3.04 ± 0.51 %ID/g), and a small fraction in the tumor (0.24 ± 0.04 %ID/g).

For [^198^Au]**6b**, the highest accumulation was found in the spleen (14.59 ± 1.75 %ID/g), followed by the liver (5.28 ± 0.35 %ID/g), the pancreas (3.88 ± 0.91 %ID/g), and a small fraction in the tumor (0.25 ± 0.10 %ID/g).

**Figure 6:**
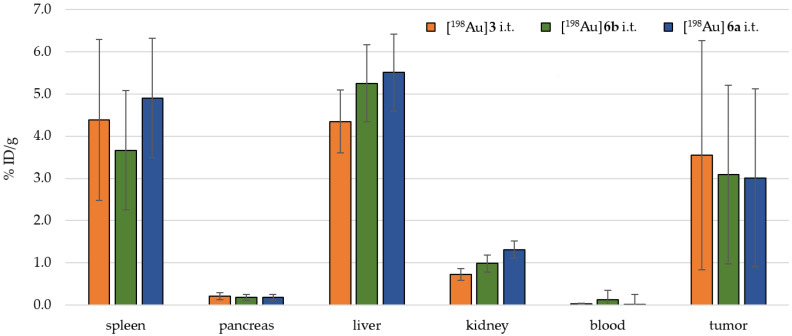
Ex vivo biodistributions of [^198^Au]**3** (orange), [^198^Au]**6a** (blue), and [^198^Au]**6b** (green) for i.t. application.

Figure 6 shows the biodistribution for different [^198^Au]AuNPs found for i.t. application. For [^198^Au]**3**, the highest accumulation was found in the liver (4.35 ± 0.74 %ID/g), the spleen (4.39 ± 1.91 %ID/g), and the tumor (3.09 ± 2.11 %ID/g), whereas in the muscle, 0.08 ± 0.02 %ID/g was found.

For [^198^Au]**6a**, the highest accumulation was found in the liver (5.51 ± 0.85 %ID/g), the spleen (4.91 ± 1.59 %ID/g), and the tumor (3.01 ± 0.76 %ID/g), whereas in the muscle, 0.08 ± 0.01 %ID/g was found.

For [^198^Au]**6b**, the highest accumulation was found in the liver (5.25 ± 0.91 %ID/g), the spleen (3.67 ± 1.41 %ID/g), and the tumor (3.09 ± 2.11 %ID/g), whereas in the muscle, 0.08 ± 0.02 %ID/g was found.

**Figure 7:**
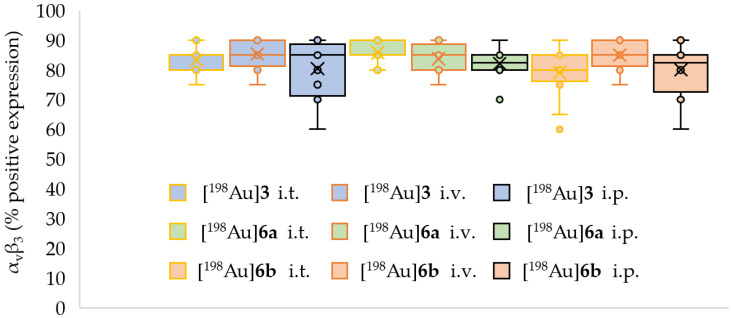
Box-plot diagram for the statistical evaluation of α_v_β_3_ expression in 54 tumors treated with [^198^Au]AuNPs showing minima/maxima (–), upper/lower quantile (o), and median (×).

After the ex vivo biodistribution, the tumors were deep-frozen and evaluated for α_v_β_3_ expression (Figure 7 and Figure A4, Table A4). It was found that all tumors had similar high α_v_β_3_ expression without significant differences. Additionally, staining for caspase3, CD31, and HE were performed and showed no abnormality.

## 3. Discussion

### 3.1. In Vitro Experiments

c(RGDfK) is a potent, selective antagonist of α_v_β_3_, a key mediator of tumor angiogenesis and cell survival [48]. Radiolabeled RGD derivatives have been extensively employed as imaging probes to visualize angiogenic vasculature in tumors [46]. Multivalent presentation of RGD motifs markedly enhances tumor targeting and retention through avidity effects. Accordingly, gold nanoparticles (AuNPs) densely functionalized with multiple c(RGDfK) ligands are expected to achieve superior tumor accumulation [38,47], thereby improving the efficacy of both targeted therapy and diagnostic imaging [36]. Methods for preparing [^198^Au]AuNPs have been previously reported in the literature [17,33,36]. The conventional synthesis route involves neutron activation of gold foil, which is then dissolved in aqua regia. Subsequent steps include nanoparticle formation and functionalization with target-specific ligands. However, all these steps are performed using radioactive ^198^Au, resulting in increased radiation exposure for personnel and further generate considerably more radioactive waste.

Tumor-specific AuNPs exhibiting high target avidity and colloidal stability were synthesized first, with neutron activation reserved as the final step to minimize operator dose and streamline the overall workflow—key requirements for clinical translation. The key challenge was to synthesize α_v_β_3_-targeted AuNPs that retain colloidal stability and ligand-shell integrity during neutron activation. AuNPs **6a** and **6b** met this requirement and exhibited superior α_v_β_3_ avidity compared with the monomeric c(RGDfK) peptide.

In order to assess the therapeutic efficacy of the [^198^Au]AuNPs, a colony formation assay was performed. The activity and incubation time required to achieve relevant doses between 5 and 10 Gy were calculated for monolayer cell cultures in 24-well plates (Formula 1). To reach these doses, concentrations more than 10 times higher than the IC_50_ for AuNP-RGD **6a** and **6b** had to be used within 96 h of incubation. Consequently, cell viability is expected to be severely impaired at such high concentrations of [^198^Au]**6** in cell survival experiments, as the RGD antagonist may interfere with angiogenesis and thus compromise cell viability [48]. Nevertheless, reference cell survival experiments demonstrated a significant effect on U-87 MG cells, with a survival fraction as low as 40.6 ± 7.3% at 10 Gy even for non-specific [^198^Au]**3** without RGD coating. This suggests that the combination of β^−^-emission from ^198^Au and the antagonistic effect of RGD could dramatically reduce the therapeutically relevant dose of RGD functionalized [^198^Au]AuNPs. In the cell experiments addressing this hypothesis, a slightly lower but significant effect was observed when both noxae were applied at 10 Gy. The effect was higher for [^198^Au]**6b** with lower RGD-loading (sf_[198Au]**6b**_ = 32.7 ± 6.1% vs. sf**_6b_** 57.3 ± 3.9%, Δ_sf_ = 24.6%) and lower for [^198^Au]**6a** with high RGD-loading (sf_[198Au]**6a**_ = 34.7 ± 8.6% vs. sf**_6a_** 41.6 ± 2.7%, Δ_sf_ = 6.9%), since the high RDG-loading of **6a** itself had already a higher cytotoxic effect compared to **6b**.

A limitation for the evaluation of cell survival was the pH of the cell media, as substantial amounts of Asc were introduced to the cell media together with the [^198^Au]AuNPs. Asc hat to be used as stabilizing agent during neutron irradiation of the AuNPs and could therefore not be excluded from the cell experiments. Therefore, the effect of Asc on the pH of cell media and consequently on cell survival was tested without and with AuNPs. However, up to a concentration of 7.1 mM Asc, the pH of the cell media was 6.6, and no significant effect on cell survival was observed. This concentration of Asc resulted in a concentration of AuNPs between 1.0 and 1.4 µM. At higher concentrations of Asc, the pH was lower than 6.6, resulting in significantly lower cell survival. Therefore, Asc concentration of 7.1 mM was the limit for the evaluation of the cell survival for [^198^Au]AuNPs.

### 3.2. In Vivo Preclinical Experiments

Therapeutic in vivo experiments were performed at U-87 MG xenograft models. Three different modes of injection (i.v., i.p., and i.t.) were evaluated to find the optimal application method for the [^198^Au]AuNPs. During the therapeutic application, planar imaging of the distribution of the [^198^Au]AuNPs in the animals revealed already that for i.v. application, most of the activity remained within the tail vein (~70%), and the rest (~30%) was found in the region of the liver of the animals. For the i.p. application, most of the activity remained within the liver (~60%) and stomach (~40%) region of the animals. However, for i.t. application, a fraction of the activity remained within the tumor (~10%), whereas the rest was found in the liver region (~90%) compared to i.v. and. i.p. application.

These observations were afterwards confirmed by ex vivo biodistribution of the animals (Figure 4, Figure 5 and Figure 6): For i.v. application, the activity had an accumulation pattern following tail > liver > spleen >>> tumor. For the i.p. application, the activity had an accumulation pattern following spleen > liver > pancreas, but a small fraction was found within the tumor (0.25 ± 0.10 %ID/g), with a tumor-to-muscle ratio of 4–6:1. In consequence, for i.t. application, a higher fraction of the activity was retained in the tumor (3.09 ± 2.11 %ID/g) compared to i.v. and i.p. application, with an accumulation pattern following liver > spleen > tumor >>> muscle.

Additionally, pathologic staining experiments on the α_v_β_3_-expression of the tumors were performed (Table A4). All tumors exhibited similar high expression of α_v_β_3_. Therefore, the α_v_β_3_-specific AuNPs should have accumulated in the tumors. Since there was no or very low tumor accumulation observed by i.v. and i.p. application, the [^198^Au]AuNPs must have undergone degradation in the blood before they could reach the tumor site. Therefore, the PEG-shell of the AuNPs was ineffective in hindering the AuNPs against the formation of protein coronae and other effects like opsonization.

An unconfirmed explanation for the discrepancies between higher cell accumulation for targeted AuNPs in vitro compared to non-targeted AuNPs and no significant tumor accumulation in vivo for both i.v. and i.p. application for targeted and non-targeted AuNPs might be the interaction with macrophages via opsonization [18]. This would lead to a fast immobilization of the AuNPs in vivo and the observed accumulation pattern. The elevated splenic accumulation following intraperitoneal (i.p.) administration likely reflects AuNPs bound to circulating blood cells, which are preferentially filtered and degraded in the spleen, whereas macrophage-associated nanoparticles are primarily sequestered and cleared by the liver. Staining experiments of the macrophages were not performed in this work, so this explanation remains a hypothesis.

**Figure A2:**
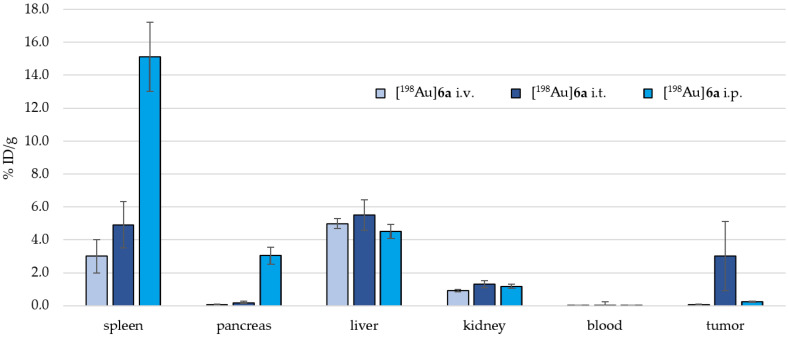
Ex vivo biodistribution of the organs of interest for different injection routes of [^198^Au]**6a**.

**Figure A3:**
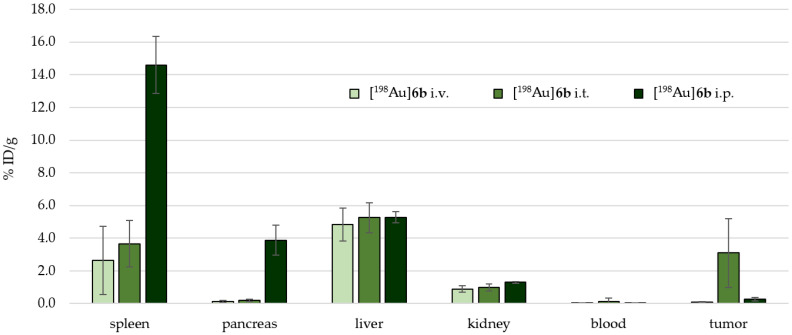
Ex vivo biodistribution of the organs of interest for different injection routes of [^198^Au]**6b**.

**Figure A4:**
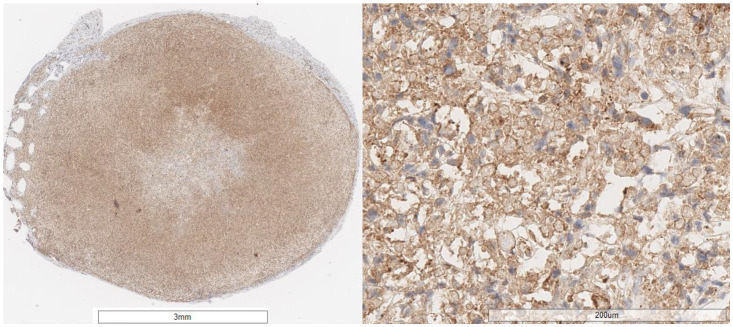
Typical pathological staining for α_v_β_3_ expression of the treated tumors.

The most promising route for application of [^198^Au]AuNPs in this work was i.t., since other injection methods revealed no significant tumor accumulation (see also Figure A1, Figure A2 and Figure A3). Lower OAR accumulation (e.g., spleen 3.7–4.9 %ID/g, liver 4.3–5.5 %ID/g) compared to i.v. and i.p. and a high tumor retention (mean 3.0–3.5 %ID/g) might pave the way for further clinical application in very special scenarios. However, it must be mentioned that the simultaneous high accumulation in the liver and spleen for i.t. application further limits the therapeutic application. By using Formula 1, doses could be calculated for a known volume of organs of interest. While tumors could receive ~5–10 Gy because of their smaller size, the liver and spleen could still receive ~2–8 Gy each due to the high-energy β^−^ particles and a 412 keV γ photon that contributes to a whole-body dose. This suggests a high dose to OARs.

I.t. application of [^198^Au]AuNPs together with external irradiation is discussed as the most effective therapeutic concept [49], and the herein developed AuNPs seem to confirm this statement. Since AuNPs are also discussed as radiosensitizer for external radiation therapy, also non-radioactive targeted AuNPs might already be useful in cancer therapy [49]. Further, other groups found i.t. application as an effective application route revealing 85% tumor retention of [^198^Au]AuNPs after 24 h in vivo [50]. Functionalized [^198^Au]AuNPs for treatment were recently evaluated in vitro on HER2-positive tumor spheroids, revealing promising therapeutic effectiveness for breast or ovarian cancers [51]. Recently, nanobrachytherapy of glioma with ^186^Re-nanoliposomes [52,53] showed promising results and might be considered as a reasonable application for [^198^Au]AuNPs as well.

In the past, AuNPs as radiopharmaceuticals were discussed as controversial [54,55], since their size of 2–5 nm might still be too large for empowering the tumor specificity of targeting vectors at their surface. Overcoming the cytotoxic effect [56,57] by using a PEG-shell was the first step towards in vivo applications [15,16]. Additionally, the PEG shell is preventing the AuNPs from forming a protein corona in vivo. In vitro evaluations of targeted AuNPs show promising results, as they constantly demonstrated lower IC_50_ values and thus higher avidities compared to the monomeric peptide lead. The cell media (EMEM) might interact with the AuNPs. Therefore, only 1% FCS was added during incubation with AuNPs, only leading to low immobilization of the AuNPs in vitro.

The molar mass of targeted AuNPs can be calculated and estimated by TGA [20]. In comparison to single molecules, which have a constant molar mass, the molar masses of AuNPs can differ from particle to particle due to their size distribution. Therefore, the IC_50_ values have to be evaluated carefully. For example, 20 RGD ligands at the surface of one AuNP would have a mass of around 12 kDa. The residual molar mass of 240–250 kDa results from the AuNP and the non-specific PEG-ligands. The calculated IC_50_ values with the whole molar mass of **6a** (262 kDa) or **6b** (257 kDa) are significantly lower compared to a single RGD peptide. However, if the molar mass of the RGD ligands only would be used for calculation of the IC_50_ values (as example, one could think about a 20-RGD-multimer peptide), a much higher IC_50_ value compared to single RGD peptide would be found. As a consequence, the calculation of IC_50_ values in general might have to be reconsidered, in the way only the molar mass of the sum of the specific ligands should be used for calculation of the IC_50_ values for targeted nanoparticles, as thinking of it like a multimer of 20 targeting molecules. However, other groups circumvented the exact determination of molar mass of AuNPs by TGA by giving the IC_50_ values in µg/mL and using the whole applicated mass of AuNPs for calculation of the competitive displacement of ^125^I-Tyr^4^BBN [18].

In conclusion, although the intact AuNP enables IC_50_ determination with high avidity in ^125^I-echistatin displacement assays, drug–receptor affinity alone does not fully predict its in vivo biodistribution. Due to their properties, the AuNPs are quite susceptible to being taken up by macrophages, lymph nodes, spleen, etc., and thus can be removed from circulation. Macrophages might detect AuNPs as debris very efficiently and could be eliminated from the blood very quickly [58]. That might be the reason for the high accumulation in the spleen and liver seen in in vivo experiments of this work and by other groups [18]. Maybe the ligand exchange for a higher density of targeted surface molecules would lead to better performing AuNPs in vivo. Taking advantage of the macrophage–AuNP interaction, it is discussed to target the macrophages directly by nanoparticles to indirectly detect tumors or inflammation in vivo [58]. For imaging of tumors, this could be a clever solution, but it remains questionable whether this method could also be used for therapy of tumors. However, other diseases like atherosclerotic cardiovascular disease [59] or autoimmune diseases [12] might be treated by this method.

Over the past five years, only a handful of studies have explored the therapeutic potential of [^198^Au]AuNPs. Although in vitro data are encouraging [51], the clinical translation of [^198^Au]AuNPs as targeted radiopharmaceuticals remains elusive. Nevertheless, AuNPs continue to advance toward clinical use—as drug-delivery vehicles [13], photothermal therapy enhancers [60], and even direct chemotherapeutic agents against select ovarian pathologies [10,11].

## 4. Materials and Methods

### 4.1. General Procedures

All reagents and solvents were purchased from commercial suppliers (Merck, Darmstadt, Germany) and were used without further purification. NMR spectra were recorded on a 300 MHz Varian Mercury Plus and a 500 MHz Varian NMR System spectrometer (Palo Alto, CA, USA). Chemical shifts (*δ*) are given in ppm and are referenced to the residual solvent resonance signals relative to (CH_3_)_4_Si (^1^H, ^13^C). Mass spectra were obtained on a Bruker Daltonics microflex MALDI-TOF mass spectrometer (Bremen, Germany) and HR-ESI-MS spectra on a Thermo Finnigan LTQ FT Ultra Fourier Transform Ion Cyclotron Resonance spectrometer (Dreieich, Germany). Preparative column chromatography was performed on Merck silica gel 60. When applicable, purity was determined by high-performance liquid chromatography (HPLC). The purity of all final compounds was 95% or higher. HPLC was performed on a Dionex UltiMate 3000 HPLC system (Thermo Scientific, Dreieich, Germany), equipped with a reverse phase column (Analytical: Chromolith RP-18e; 100 × 4.6 mm plus a guard column 5 × 4.6 mm; semipreparative: Chromolith RP-18e; 100 × 10 mm plus a guard column 10 × 4.6 mm (Merck, Darmstadt, Germany), and a UV-diode array detector (210 nm, 254 nm). The solvent system used was a gradient of acetonitrile:water (containing 0.1% TFA) (0–5 min: 0–100% MeCN) at a flow rate of 4 mL/min, unless otherwise stated. Purification of AuNPs was performed by dialysis (tubes with molecular weight cut-off of 14,000 g/mol, Visking, Roth, Karlsruhe, Germany) against distilled water and by size exclusion chromatography using Sephadex G25 PD10 columns and distilled water as eluent. The purity and stability of AuNPs/[^198^Au]AuNPs were investigated by size exclusion HPLC using a Phenomenex PolySep™-SEC GFC-P 4000, LC column 300 × 7.8 mm and a 35 mm PolySep guard column with water (0.8 mL/min) as eluent on a Thermofisher Ulti HPLC system. A brief description of the AuNP syntheses and a detailed description of the competitive displacement assays against [^125^I]I-echistatin as the competitor for the determination of IC_50_ values can be found in a previous publication [44].

### 4.2. Determination of the Number of Ligands on the Surface of the AuNPs

The thermogravimetric analyses were performed using a Mettler Toledo TGA 2 STAR^e^ system. AuNPs (1–2 mg) were weighed into 70 µL aluminum oxide crucibles (Mettler Toledo, Gießen, Germany) and heated from 25–750 °C (10 K/min) in a stream of N_2_ or CO_2_ (30 mL/min). The loading of the different AuNPs is shown in Table 1 and was calculated from the different mass losses, which increase as the AuNPs are functionalized. Therefore, the amount of different ligands per particle can be calculated according to the formula of Zhu et al. [43]. Since the AuNPs have an average diameter of ~3 nm, the calculated amount of gold atoms is ~834 Au atoms per nanoparticle. This gives a molecular weight of an AuNP of 164,298 g/mol. Using TGA, the following ligand numbers were determined:

- The mass loss of the AuNP **3** was ~33.27%, which results in ~240 PEG ligands on the AuNP surface, M ~ 246 kDa.
- The mass loss of AuNP-PEG-RGD_high_ **6a** was ~37.1%, and the RGD accounts for ~4% mass loss (~24 RGD ligands per AuNP). Therefore, the molar mass for AuNP-RGD_high_ **6a** was calculated to be ~262 kDa.
- Furthermore, the AuNP-RGD_low_ **6b** contained ~18 RGD ligands, ~257 kDa.

### 4.3. Neutron Irradiation Experiments

Production of [^198^Au]AuNPs by neutron activation of 0.05–15.5 mg AuNPs was performed in a pneumatic transfer tube for 1–2 h at 100 kW with a thermal neutron flux of 1.6 × 10^12^ cm^−2^·s^−1^ in the TRIGA research reactor (Mainz, Germany). For calibration of the dose calibrator, ISOMED 2010 (NUVIA Instruments, Dresden, Germany) 12.7 mg solid Au was irradiated for 80 min to produce 87 MBq (calculated) [^198^Au]Au with a measured dose rate of 57 µSv/h. Next, 26 h later, the activity was measured with the dose calibrator, and 60 MBq was obtained (using the ^137^Cs-channel, 66 MBq calculated). In addition, the solid [^198^Au]Au (40 MBq, 42 calc.) was carefully dissolved two days after in 2 mL aqua regia at 50 °C within 15 min in order to find the correct calibration factors of the dose calibrator for different volumes in vials and syringes.

Irradiation of AuNPs was performed under optimized conditions with a thermal neutron flux of 1.6 × 10^12^ cm^−2^·s^−1^ in 2 mL 10% EtOH/H_2_O and 25 mg ascorbic acid as stabilizer against radiolysis [61]. In the experiment, neutron activation of 4.45–10.10 mg AuNPs **3** and **6** for 2 h produced 65–147 MBq [^198^Au]**3** and [^198^Au]**6**. The production of ^198^Au was confirmed by gamma spectroscopy, which found up to three gamma lines at 411 keV (95.6%), 676 keV (0.8%), and 1088 keV (0.2%).

### 4.4. Colony Formation Assay

Glioblastoma astrocytoma (U-87 MG) tumor cells (HTB-14, ATCC^®^, Manassas, VA, USA) were cultivated with EMEM medium (ATCC^®^, Manassas, VA, USA), supplemented with 10% FCS and 1% penicillin–streptomycin (10,000 U/mL) at 37 °C in a humidified CO_2_ (5%) atmosphere. Three days before the experiments, U-87 MG cells were harvested, and 150,000 cells were seeded in 24-well plates. U-87 MG cells were incubated for 96 h in the presence of the α_v_β_3_-specific non-radioactive AuNPs or 1–2 MBq [^198^Au]AuNPs to achieve the calculated doses of 5–10 Gy. As an incubation medium, EMEM supplemented with 1% FCS and 1% penicillin–streptomycin (10,000 U/mL) was used. After incubation, the cell medium was removed, the cells were washed and harvested, and a colony formation assay was performed in triplicate for each irradiation point with 1000 cells per well in a 6-well plate. Colonies were cultured in the cell medium (EMEM, 10% FCS, 1% penicillin–streptomycin) for 28 days, fixed with 2 mL 4% formaldehyde in PBS for 15 min, and incubated with 2 mL 0.5% crystal violet dye solution for 30 min. Afterwards, colonies were washed with distilled water, dried, and counted by light microscopy. Colonies of more than 50 cells were considered viable, and the plating efficiency for each sample was estimated based on the initial number of cells seeded. Clonogenic cell survival was calculated as the relative plating efficiency of treated versus untreated samples. Triplicate samples were prepared for each treatment and experimental condition.

### 4.5. In Vivo Experiments

The in vivo proof-of-concept was performed using female athymic nude mice (Rj:ATHYM-*Foxn1^nu/nu^*) obtained from Janvier Labs (approval number 35-9185.81/G-255/22). A total of 5 × 10^6^ U-87 MG cells (100 µL in PBS, unsieved) were inoculated subcutaneously in the right thigh when the mice were 6 weeks old. Mouse health and tumor growth were checked daily until the tumor reached a diameter of 5 mm (1 week for U-87 MG). After the tumors reached a sufficient size for imaging, the AuNPs were injected i.v. into the tail vein, i.p. into the cavity of stomach, or i.t., and their distribution in vivo was monitored after 1, 3, and 7 days via optical imaging (using a radiographic phosphor screen activated by the gamma radiation of ^198^Au), followed by an X-ray imaging (0.8 mm filter, 45 kV, 5 s) (In-Vivo Xtreme, Bruker, Ettlingen, Germany). After the last time point, animals were sacrificed, the organs were harvested, and their activity was measured ex vivo. The region-of-interest (ROI) was drawn by hand on the organs for calculation of the uptake of the AuNPs in the respective organs. Bruker molecular imaging software MI SE (version 7.1.3.20550) was used for the fusion of the images. All injections and measurements with mice were performed under anesthesia (2–3% isoflurane/O_2_, 2–3 mL/min).

## 5. Conclusions

In this work, α_v_β_3_-specific RGD-coated AuNPs with enhanced target avidity compared to the monomeric α_v_β_3_-specific RGD reference peptide were successfully synthesized and fully characterized. This proof-of-concept study aimed to evaluate the feasibility of activating AuNPs while preserving the integrity of their ligand shell and organic coating. Irradiation experiments confirmed the stability of [^198^Au]AuNPs with dithiol ligands after neutron activation. In vitro experiments evaluating the therapeutic effect of [^198^Au]AuNPs on U-87 MG cell survival revealed a significant impact on cell death, with RGD-functionalized [^198^Au]AuNPs showing a higher efficacy compared to the non-targeted analogs.

Encouraged by these results, the preclinical therapeutic application of [^198^Au]AuNPs in vivo was evaluated using different modes of injection (local vs. systemic). The [^198^Au]AuNPs were assessed in vivo in human U-87 MG tumor xenografts with three distinct application routes. Intravenous (i.v.) and intraperitoneal (i.p.) administration revealed no significant target-specific tumor accumulation but high accumulation in off-target organs. Consequently, the systemic application of the developed [^198^Au]AuNPs does not seem to be feasible. In contrast, intratumoral (i.t.) injection resulted in relatively stable tumoral retention of the [^198^Au]AuNPs, although a considerable accumulation in non-target tissues was still observed, albeit at lower levels compared to i.v. and i.p. administration.

Importantly, no significant difference in target-specific tumor accumulation between RGD- and non-RGD-functionalized [^198^Au]AuNPs was observed in vivo for all three application routes. These results support the technical feasibility and proof-of-concept for i.t. application, but a clinical translation of the developed [^198^Au]AuNPs is limited due to their biodistribution properties. However, they may be useful for specialized treatments involving i.t. application.

## Figures and Tables

**Table 1:** Calculated number of ligands and resulting molecular mass of the AuNPs.

AuNP Sample	Description	Number of Ligands	Molecular Mass [kDa]
**3**	AuNP-dithio-PEG	240× **2**	246
**6a**	AuNP-dithio-PEG-RGD_high_	218× **2**, 24× **5**	262
**6b**	AuNP-dithio-PEG-RGD_low_	220× **2**, 18× **5**	257

**Table 2:** Activity yields for neutron irradiation of different AuNPs.

Sample	Amount AuNP	Amount of Gold	Calculated ^198^Au Activity After Irradiation	Obtained Activity *
gold	-	12.7 mg (100%)	87 MBq	60 MBq *
**3**	15.30 mg	10.10 mg (66%)	102 MBq	144.5 MBq **
**3**	7.11 mg	4.69 mg (66%)	50.6 MBq	65.9 MBq **
**6a**	14.62 mg	9.21 mg (63%)	103 MBq	146.8 MBq **
**6a**	7.07 mg	4.45 mg (63%)	50.8 MBq	65.4 MBq **
**6b**	14.84 mg	9.65 mg (65%)	103 MBq	140.7 MBq **
**6b**	7.00 mg	4.55 mg (65%)	50.8 MBq	69.5 MBq **

* Obtained activity measured 26 h after irradiation, decay corrected activity 65.6 MBq. ** Obtained activity measured 2–3 h after irradiation.

**Table 3:** Concentration of AuNP solutions for irradiation and the corresponding pH within the cell media to determine the influence of Asc towards cell survival.

Concentration of AuNP 3	Concentration of Ascorbic Acid	pH (AuNP/AS)	pH (AuNP/AS in Medium)
0 µM (medium only)	-	-	7.9
10.1 µM (stock)	70.97 mM	3.5	-
0.254 µM	1.77 mM	4.0	7.2
0.507 µM	3.55 mM	3.8	7.1
1.01 µM	7.10 mM	3.7	6.6
2.03 µM	14.19 mM	3.5	5.4
Asc only	14.19 mM	3.5	5.1

## Data Availability

The original contributions presented in this study are included in the article. Further inquiries can be directed to the corresponding author.

## Abbreviations

Asc (ascorbic acid), AuNPs (gold nanoparticles), c (concentration), IC_50_ (AuNP-concentration displacing 50% of the radioligand ^125^I-echistatin from the U-87 MG cells), i.p. (intraperitoneal), i.t. (intratumoral), i.v. (intravenous), OARs (organ at risk), SD (standard deviation), %ID/g (percentage of whole injected dose of [^198^Au]AuNP).

## Appendix A

### Appendix A.1. Organic Syntheses

- c(RGDfK) [62]

The peptide c(RGDfK) c(Arg-Gly-Asp-D-Phe-Lys) was synthesized according to standard protocols by solid-phase peptide synthesis on solid support using the Fmoc-strategy on H-Asp(*t*Bu)-2-chlortrityl resin (loading 0.73 mmol/g, 137 mg, 0.1 mmol). For amino acid conjugation, HBTU (*N*,*N*,*N*′,*N*′-tetramethyl-*O*-(*1H*-benzotriazol-1-yl)uronium hexafluorophosphate) (3.9 eq., 0.39 mmol, 148.7 mg), Fmoc-protected amino acids (4.0 eq., 0.4 mmol), and DIPEA (4.0 eq., 0.4 mmol, 68 µL) were used in DMF as solvent. Each amino acid was coupled for 45 min. After coupling of the last amino acid and Fmoc-removal with 50% of piperidine solution in DMF, the linear protected peptide was cleaved from the resin using 1% TFA in CH_2_Cl_2_. After removal of the volatile components of the mixture, the crude intermediate was isolated and dissolved in dry DMF (85 mL). After the addition of DIPEA (3.5 eq., 0.35 mmol, 59.5 µL), the solution was cooled to 0 °C, and DPPA (1.25 eq., 0.125 mmol, 26.9 µL) was added. The reaction was allowed to warm to ambient temperature and stirred for 3 days until the cyclization was complete. The volatile components of the mixture were evaporated under reduced pressure, and the residue was treated with a mixture of TFA/TIS 97.5:2.5 for 3 h to completely deprotect the peptide. The crude product was precipitated in cold diethyl ether and washed twice with diethyl ether and dried under reduced pressure. The product was purified by semi-preparative HPLC and lyophilized to give a colorless solid (yield: 88.79%, 53.6 mg, 0.089 mmol). HR-ESI-MS (*m*/*z*) for [M + H]^+^ (calculated): 604.3 (604.3). MALDI-MS (*m*/*z*) for [M]^+^ (calculated): 603.7 (603.3).

- TA-NHS **1** [63]

Thioctic acid (TA) (1 eq., 2.425 mmol, 0.50 g) was dissolved in acetone (12.5 mL) under an argon atmosphere. *N*,*N*-Disuccinimidyl carbonate (1.25 eq., 3.031 mmol, 0.78 g) and DIPEA (1.25 eq., 3.031 mmol, 0.5 mL) were added, and the mixture was stirred overnight at room temperature. After removal of acetone under reduced pressure, the residue was dissolved in 10 mL of water and DCM (1:1). The aqueous phase was removed, and the organic phase was washed twice with 4 mL water. After drying the organic phase with MgSO_4,_ the solvent, DCM, was removed, and TA-NHS was obtained as a yellowish solid. (78.01%, 574 mg, 1.89 mmol). ^1^H-NMR (300 MHz, DMSO-*d*_6_) *δ* = 3.67–3.58 (m, 1H, *H*-3), 3.24–3.08 (m, 2H, *H*-5), 2.81 (s, 4H, *H*-15, *H*-16), 2.69 (t, ^3^*J* = 7.2 Hz, 2H, *H*-9), 2.47–2.37 (m, 1H, *H*-4), 1.94–1.83 (m, 1H, *H*-4), 1.77–1.42 ppm (m, 6H, *H*-6, *H*-7, *H*-8).

- TA-PEG_3_-OH **2**

TA-NHS dissolved in 3 mL DCM (1.2 eq., 0.96 mmol, 0.29 g) was added to a solution of H_2_N-PEG_3_-OH (1 eq., 0.80 mmol, 0.12 g) in DCM (1 mL). After, DIPEA (4 eq., 3.2 mmol, 545 µL) was added, and the mixture was stirred overnight at ambient temperature. The solvent was removed under reduced pressure, and the crude product was purified by semi-preparative HPLC. Finally, the product TA-NH-PEG_3_-OH was isolated as yellowish viscous liquid (yield: 65.04%, 180 mg, 0.533 mol). ^1^H-NMR (500 MHz, DMSO-*d*_6_) *δ* = 7.89–7.77 (m, 1H, *H*-11), 3.43–3.37 (m, 12H, *H*-13, *H*-14, *H*-16, *H*-17, *H*-19, *H*-20), 3.22–3.15 (m, 2H, *H*-5), 2.87–2.71 (m, 2H, *H*-3, *H*-21), 2.13–2.03 (m, 2H, *H*-9), 1.99–1.81 (m, 2H, *H*-4), 1.60–1.21 ppm (m, 6H, *H*-6, *H*-7, *H*-8). HR-ESI-MS (*m*/*z*) for [M + H]^+^ (calculated): 338.1 (338. 1), [M + Na]^+^ (calculated): 360.1 (360.1). MALDI-MS (*m*/*z*) for [M]^+^ (calculated): 337.3 (337.1), [M + H]^+^ (calculated): 338.1 (338. 1).

- TA-PEG_4_-COOH **4**

TA-NHS dissolved in 2 mL DCM (1.1 eq., 0.88 mmol, 0.27 g) was added to a suspension of H_2_N-PEG_4_-COOH (1 eq., 0.80 mmol, 0.12 g) in DMF (2 mL). DIPEA (4 eq., 3.2 mmol, 545 µL) was added, and the mixture was stirred overnight at ambient temperature. The solvent was removed under reduced pressure, and the crude product was purified by semi-preparative HPLC. After lyophilization the product TA-NH-PEG_4_-COOH was obtained as colorless solid (yield: 48.92%, 160.3 mg, 0.39 mmol). HR-ESI-MS (*m*/*z*) for [M + H]^+^ (calculated): 410.1 (410.1), [M + Na]^+^ (calculated): 432.1 (432.1). MALDI-MS (*m*/*z*) for [M]^+^ (calculated): 409.4 (409.1), [M + K]^+^ (calculated): 447.4 (448.1).

- TA-PEG_4_-c(RGDfK) **5**

TA-NH-PEG_4_-COOH dissolved in 0.5 mL of DMF (1.1 eq., 0.055 mmol, 22.4 mg) was added to a solution of PyBOP (1.9 eq., 0.094 mmol, 49.1 g) in 0.5 mL of DMF. DIPEA (3 eq., 0.149 mmol, 26 µL) was added, and the mixture was stirred at ambient temperature until the reaction was completed. DMF was then removed under reduced pressure, and the crude product was purified by semi-preparative HPLC. After lyophilization, the product TA-NH-PEG_4_-c(RGDfK) was obtained as colorless solid (yield: 28.91%, 14.3 mg, 0.014 mmol). HR-ESI-MS (*m*/*z*) for [M + H]^+^ (calculated): 995.4 (995.4). MALDI-MS (*m*/*z*) for [M]^+^ (calculated): 994.8 (994.4), [M + K]^+^ (calculated): 1033.7 (1033.4)

- AuNP-dithio-PEG_3_-OH **3** [41]

Hydrogen tetrachloroaurate (1 eq., 0.525 mmol, 207 mg) was dissolved in 205 mL MeOH to give a bright yellow solution, and under stirring, a solution of TA-NH-PEG_3_-OH dissolved in 205 mL of MeOH was added and stirred for 2 h until the reaction color became nearly colorless. A solution of sodium borohydride dissolved in 20.5 mL of water was quickly added to the reaction. The solution immediately turned black. The reaction mixture was stirred overnight, MeOH was removed under reduced pressure, and the residue was redissolved in 9 mL of trace pure water and dialyzed in distilled water for 4 days. After lyophilization, TA-AuNP was obtained as black powder. (39.47%, 180.7 mg)

- AuNP-PEG-RGDs by ligand exchange

General procedure for the preparation of RGD-decorated AuNPs: Briefly, the functionalization of AuNPs **6** was performed by a place-exchange reaction with TA-PEG_4_-c(RGDfK) **5**. TA-PEG_4_-c(RGDfK) **5** was dissolved in a mixture trace pure H_2_O:MeOH (1:1) and was added to AuNPs **6** in 2 mL trace pure H_2_O and stirred overnight. Purification of AuNPs was performed in two steps: first, the AuNP solution was transferred into a Visking cellulose dialysis tube (molecular cut-off 14,000 Da) with 3 × 1 mL trace pure water and dialyzed in distilled water for 4 days, and second, the AuNP solution was eluted by size-exclusion chromatography using Sephadex G25 PD10 columns and distilled water. The AuNPs were then lyophilized and obtained as a black powder.

- AuNP-dithio-PEG-RGD_high_ **6a**

TA-PEG_4_-c(RGDfK) **5** (8 mg, 8.04 µmol) was dissolved in 0.4 mL of trace pure H_2_O:MeOH (1:1) and was added to AuNP **3** (20 mg) in 2 mL of trace pure H_2_O and stirred overnight. After purification and lyophilization, 20.8 (97.5%) of **8a** was obtained as black powder. ^1^H-NMR (500 MHz, DMSO-*d*_6_) *δ* = 8.02–7.59 (m, 7H, *H*-11, *H*-25, *H*-29, *H*-34, *H*-39, *H*-43, *H*-47), 7.22–7.16 (m, 5H, *H*-64, *H*-65, *H*-66, *H*-67, *H*-68), 6.65 (s, 2H, *H*-57), 4.77–4.43 (m, 1H, *H*-u), 3.61–3.35 (m, 10H, *H*-13, *H*-14, *H*-16, *H*-17, *H*-19, *H*-20), 3.22–3.00 (m, 4H, *H*-22, *H*-23), 2.92–2.69 (m, 1H, *H*-3), 2.13–1.95 (m, 2H, *H*-5), 1.76–0.73 ppm (m, 10H, *H*-4, *H*-6, *H*-7, *H*-8, *H*-9).

- AuNP-dithio-PEG-RGD_low_ **6b**

TA-PEG_4_-c(RGDfK) **5** (4 mg, 4.02 µmol) was dissolved in 0.4 mL of trace pure H_2_O:MeOH (1:1) and was added to AuNP **3** (20 mg) in 2 mL of trace pure H_2_O and stirred overnight. After purification and lyophilization, 19.7 mg (95.1%) of **8b** was obtained as black powder.

### Appendix A.2. Electron Microscopy

AuNP samples were diluted at will in deionized water (fade red solution), then particles were adsorbed onto glow-discharged carbon-coated EM grids and directly observed by TEM (Zeiss EM912, Carl Zeiss, Oberkochen, Germany). Images were digitally captured with a CCD camera (Sharp eye, TRS, Moorenweiss, Germany). Particle number and size were measured using the FIJI software (v1.50e) [44].

**Table A1:** Gamma spectrum analysis (maximum 2 MeV) after neutron activation of ^197^Au and identified radionuclides in bold red and the identified gamma signals in bold black.

Nuclide	Half-Life	Gamma Energy [keV] Incidence [%]
^197^Au →**^198^Au** → ^198^Hg (stable)	2.6941 d	**411.80205 (95.6%)**
^23^Na → ^24^**Na** → ^24^Mg (stable)	14.956 h	**1368.625 (99.9%)** 2754.008 (99.9%)
^37^Cl (24.4% isotopic incidence) → ^38^**Cl** → ^38^Ar (stable)	37.230 min	**1642.68 (32.9%)** 2167.400 (44.0%)
^40^Ar →**^41^Ar** → ^41^K (stable)	109.61 min	**1293.64 (99.2%)**
^55^Mn →**^56^Mn** → ^56^Fe (stable)	2.5789 h	**846.7638 (98.9%)** 1810.726 (26.9%) 2113.092 (14.2%) 2523.06 (1.018%)

**Table A2:** Results of the cell survival experiments shown in Figure 2.

Type of AuNP and dose	Number of Colonys	Standard Deviation (SD)	Survival Fractions [%]	SD [%]	c AuNP [µM]	c Asc [mM]
	101 (control)	4	100.0	4.0	0	-
[^198^Au]**6a** 5 Gy	42	6	41.6	14.3	0.40	1.15
[^198^Au]**6a** 10 Gy	35	3	34.7	8.6	0.80	2.31
[^198^Au]**6b** 5 Gy	38	6	37.6	15.8	0.54	1.49
[^198^Au]**6b** 10 Gy	33	2	32.7	6.1	1.07	2.98
[^198^Au]**3** 5 Gy	42	2	41.6	4.8	0.72	1.77
[^198^Au]**3** 10 Gy	41	3	40.6	7.3	1.44	3.55
	89 (control)	4	100.0	4.5	-	-
**6a** (c like 5 Gy)	39	2	43.8	5.1	0.40	1.15
**6a** (c like 10 Gy)	37	1	41.6	2.7	0.80	2.31
**6b** (c like 5 Gy)	50	2	56.2	4.0	0.54	1.49
**6b** (c like 10 Gy)	51	2	57.3	3.9	1.07	2.98

**Table A3:** Results of the ex vivo biodistribution for 54 animals shown in Figure 4, Figure 5 and Figure 6.

							**Mean**	
**[^198^Au]3 i.v. [MBq]**	**2.37**	**7.54**	**7.29**	**2.14**	**3.45**	**1.72**	**4.09**	
**Organs**	**Mouse 01**	**Mouse 02**	**Mouse 03**	**Mouse 04**	**Mouse 05**	**Mouse 06**	**%ID/g** **n = 6**	**±SD** **n = 6**
intestine small	0.04	0.15	0.14	0.02	0.04	0.03	0.07	0.05
intestine large	0.08	0.20	0.19	0.03	0.09	0.04	0.10	0.07
stomach	0.07	0.24	0.17	0.04	0.08	0.05	0.11	0.07
spleen	2.71	7.06	6.07	1.58	3.08	1.16	3.61	2.21
pancreas	0.10	0.29	0.29	0.04	0.10	0.07	0.15	0.10
liver	5.22	4.04	3.54	4.14	4.81	1.95	3.95	1.05
kidney	0.46	0.83	0.84	0.42	0.48	0.33	0.56	0.20
blood	0.02	0.02	0.02	0.02	0.02	0.02	0.02	0.00
heart	0.14	0.66	0.57	0.11	0.19	0.12	0.30	0.23
lung	0.10	0.68	0.49	0.10	0.20	0.07	0.27	0.23
tail	5.96	0.67	2.81	7.39	7.97	5.08	4.98	2.55
brain	0.00	0.01	0.01	0.00	0.00	0.00	0.00	0.00
tumor	0.06	0.11	0.11	0.06	0.06	0.05	0.07	0.02
bone	0.42	0.83	0.90	0.23	0.53	0.46	0.56	0.23
muscle	0.04	0.21	0.09	0.04	0.06	0.03	0.08	0.06
**[^198^Au]3 i.t. [MBq]**	**4.16**	**3.95**	**4.87**	**5.73**	**3.68**	**4.03**	**4.40**	
	**Mouse 07**	**Mouse 08**	**Mouse 09**	**Mouse 10**	**Mouse 11**	**Mouse 12**	**%ID/g** **n = 6**	**n = 6**
intestine small	0.04	0.11	0.15	0.12	0.09	0.05	0.09	0.04
intestine large	0.07	0.16	0.15	0.19	0.15	0.10	0.13	0.04
stomach	0.07	0.15	0.21	0.19	0.17	0.09	0.14	0.05
spleen	1.82	3.56	7.00	6.17	5.32	2.46	4.39	1.91
pancreas	0.08	0.25	0.26	0.30	0.24	0.11	0.21	0.08
liver	3.44	4.47	4.81	4.21	5.61	3.54	4.35	0.74
kidney	0.52	0.89	0.78	0.87	0.72	0.57	0.73	0.14
blood	0.04	0.02	0.02	0.04	0.03	0.03	0.03	0.01
heart	0.15	0.45	0.52	0.59	0.42	0.22	0.39	0.16
lung	0.11	0.28	0.41	0.49	0.35	0.14	0.30	0.14
tail	0.07	0.19	0.17	0.18	0.17	0.11	0.15	0.04
brain	0.00	0.01	0.01	0.01	0.00	0.00	0.01	0.00
tumor	18.42	1.44	3.50	1.34	2.70	8.73	3.54	2.71
bone	0.37	0.70	0.83	1.00	0.89	0.58	0.73	0.21
muscle	0.07	0.11	0.08	0.08	0.09	0.05	0.08	0.02
**[^198^Au]3 i.p. [MBq]**	**5.13**	**5.20**	**5.07**		**3.68**		**4.77**	
	**Mouse 13**	**Mouse 14**	**Mouse 15**	**Mouse 16**	**Mouse 17**	**Mouse 18**	**%ID/g** **n = 6**	**n = 6**
intestine small	0.04	0.72	0.04	0.86	0.88	0.07	0.43	0.39
intestine large	0.06	0.32	0.06	0.91	0.61	0.08	0.34	0.32
stomach	0.11	0.76	0.05	0.56	0.44	0.12	0.34	0.26
spleen	0.75	6.56	1.05	11.01	10.36	0.79	5.09	4.45
pancreas	0.23	2.74	0.11	4.25	2.46	0.20	1.67	1.59
liver	1.36	4.46	1.51	6.90	5.18	1.71	3.52	2.12
kidney	0.43	0.80	0.47	1.00	0.86	0.62	0.70	0.21
blood	0.04	0.02	0.03	0.02	0.02	0.06	0.03	0.01
heart	0.07	0.19	0.06	0.28	0.30	0.09	0.16	0.10
lung	0.06	0.10	0.06	0.54	0.21	0.08	0.17	0.17
tail	0.05	0.09	0.05	0.15	0.18	0.06	0.10	0.05
brain	0.00	0.00	0.00	0.00	0.01	0.00	0.00	0.00
tumor	1.66	0.20	1.68	0.22	0.23	7.53	0.22	0.01
bone	0.16	0.53	0.15	0.66	0.84	0.23	0.43	0.26
muscle	0.03	0.05	0.03	0.06	0.07	0.07	0.05	0.02
**[^198^Au]6a i.v. [MBq]**	**3.09**	**3.40**	**4.53**	**2.65**	**3.46**	**2.84**	**3.33**	
	**Mouse 19**	**Mouse 20**	**Mouse 21**	**Mouse 22**	**Mouse 23**	**Mouse 24**	**%ID/g** **n = 6**	**n = 6**
intestine small	0.08	0.08	0.10	0.07	0.07	0.08	0.08	0.01
intestine large	0.08	0.06	0.07	0.05	0.05	0.06	0.06	0.01
stomach	0.07	0.08	0.10	0.06	0.10	0.08	0.08	0.01
spleen	2.52	4.10	4.30	1.58	2.08	3.43	3.00	1.02
pancreas	0.07	0.08	0.07	0.05	0.08	0.07	0.07	0.01
liver	5.32	4.95	4.62	4.63	5.04	5.36	4.99	0.29
kidney	0.90	0.89	0.99	0.79	0.93	0.97	0.91	0.06
blood	0.02	0.03	0.02	0.02	0.01	0.02	0.02	0.00
heart	0.15	0.20	0.17	0.19	0.24	0.22	0.20	0.03
lung	0.11	0.15	0.19	0.20	0.21	0.20	0.18	0.03
tail	9.64	6.55	7.75	7.19	6.96	6.88	7.50	1.03
brain	0.00	0.00	0.01	0.00	0.00	0.00	0.00	0.00
tumor	0.09	0.08	0.08	0.07	0.07	0.08	0.08	0.01
bone	0.49	0.54	0.58	0.47	0.54	0.50	0.52	0.04
muscle	0.03	0.04	0.02	0.03	0.06	0.05	0.04	0.01
**[^198^Au]6a i.t. [MBq]**	**4.74**	**4.30**	**4.62**	**4.42**	**4.71**	**4.89**	**4.61**	
	**Mouse 25**	**Mouse 26**	**Mouse 27**	**Mouse 28**	**Mouse 29**	**Mouse 30**	**%ID/g** **n = 6**	**n = 6**
intestine s	0.08	0.25	0.18	0.19	0.19	0.26	0.19	0.06
intestine l	0.06	0.17	0.12	0.11	0.13	0.16	0.12	0.04
stomach	0.08	0.21	0.16	0.16	0.15	0.20	0.16	0.04
spleen	2.15	4.72	5.70	5.54	4.02	7.29	4.91	1.59
pancreas	0.07	0.24	0.20	0.18	0.19	0.23	0.19	0.05
liver	3.83	6.04	5.42	6.06	5.26	6.42	5.51	0.85
kidney	0.78	1.33	1.46	1.45	1.27	1.58	1.31	0.26
blood	0.03	0.02	0.02	0.02	0.01	0.03	0.02	0.01
heart	0.24	0.85	0.70	0.66	0.70	0.88	0.67	0.21
lung	0.33	0.67	0.65	0.67	0.52	0.76	0.60	0.14
tail	0.05	0.13	0.09	0.10	0.14	0.13	0.11	0.03
brain	0.01	0.01	0.01	0.01	0.01	0.01	0.01	0.00
tumor	23.81	2.50	2.80	4.23	3.46	2.08	3.01	0.76
bone	0.44	0.55	0.68	0.96	0.64	0.82	0.68	0.17
muscle	0.07	0.09	0.08	0.06	0.09	0.10	0.08	0.01
**[^198^Au]6a i.p. [MBq]**	**5.08**	**5.05**	**5.15**	**5.16**	**5.13**	**5.08**	**5.11**	
	**Mouse 31**	**Mouse 32**	**Mouse 33**	**Mouse 34**	**Mouse 35**	**Mouse 36**	**%ID/g** **n = 6**	**n = 6**
intestine small	0.57	0.80	0.65	0.69	1.40	1.10	0.87	0.29
intestine large	1.05	1.45	1.03	0.94	0.61	0.99	1.01	0.25
stomach	1.70	0.54	0.57	0.54	0.64	0.56	0.76	0.42
spleen	19.35	13.12	13.33	14.76	14.34	15.82	15.12	2.10
pancreas	2.70	2.64	3.35	2.77	4.05	2.73	3.04	0.51
liver	5.10	4.84	4.12	4.85	4.13	4.04	4.51	0.43
kidney	1.33	1.22	1.07	1.35	1.03	1.08	1.18	0.13
blood	0.02	0.02	0.01	0.02	0.02	0.02	0.02	0.00
heart	0.28	0.25	0.23	0.22	0.24	0.23	0.24	0.02
lung	0.43	0.32	0.40	0.32	0.27	0.58	0.39	0.10
tail	0.09	0.10	0.08	0.07	0.07	0.09	0.08	0.01
brain	0.00	0.01	0.01	0.00	0.00	0.00	0.00	0.00
tumor	0.21	0.28	0.29	0.17	0.24	0.21	0.24	0.04
bone	0.62	0.64	0.50	0.21	0.46	0.40	0.47	0.15
muscle	0.05	0.05	0.04	0.05	0.03	0.04	0.04	0.01
**[^198^Au]6b i.v. [MBq]**	**3.02**	**2.20**	**1.03**	**4.81**	**2.45**	**6.98**	**3.41**	
	**Mouse 37**	**Mouse 38**	**Mouse 39**	**Mouse 40**	**Mouse 41**	**Mouse 42**	**%ID/g** **n = 6**	**n = 6**
intestine small	0.19	0.07	0.13	0.07	0.10	0.34	0.15	0.09
intestine large	0.09	0.06	0.08	0.05	0.10	0.20	0.10	0.05
stomach	0.13	0.08	0.09	0.07	0.06	0.19	0.10	0.05
spleen	2.18	2.05	1.44	1.58	1.37	7.24	2.64	2.08
pancreas	0.13	0.07	0.09	0.05	0.08	0.30	0.12	0.09
liver	5.73	4.62	6.45	3.64	4.83	3.75	4.84	1.01
kidney	0.99	0.71	1.00	0.75	0.68	1.23	0.89	0.20
blood	0.02	0.02	0.02	0.02	0.05	0.02	0.03	0.01
heart	0.38	0.13	0.20	0.16	0.21	0.67	0.29	0.19
lung	0.31	0.14	0.18	0.13	0.21	0.35	0.22	0.08
tail	6.34	10.91	12.97	7.20	8.12	1.09	7.77	3.74
brain	0.01	0.01	0.01	0.00	0.00	0.01	0.01	0.00
tumor	0.08	0.09	0.08	0.09	0.07	0.14	0.09	0.02
bone	0.61	0.54	0.87	0.30	0.47	0.91	0.62	0.21
muscle	0.05	0.02	0.05	0.12	0.02	0.11	0.06	0.04
**[^198^Au]6b i.t. [MBq]**	**4.59**	**4.10**	**4.17**	**4.54**	**4.49**	**4.41**	**4.38**	
	**Mouse 43**	**Mouse 44**	**Mouse 45**	**Mouse 46**	**Mouse 47**	**Mouse 48**	**%ID/g** **n = 6**	**n = 6**
intestine small	0.11	0.21	0.17	0.30	0.09	0.29	0.19	0.08
intestine large	0.08	0.16	0.13	0.18	0.08	0.17	0.13	0.04
stomach	0.08	0.17	0.15	0.21	0.08	0.25	0.16	0.06
spleen	2.00	3.94	3.62	3.90	2.24	6.30	3.67	1.41
pancreas	0.09	0.25	0.20	0.27	0.09	0.19	0.18	0.07
liver	3.92	5.06	5.71	5.75	4.40	6.68	5.25	0.91
kidney	0.68	1.01	0.98	1.20	0.77	1.25	0.98	0.21
blood	0.03	0.63	0.02	0.02	0.03	0.02	0.13	0.22
heart	0.24	0.62	0.52	0.68	0.31	0.80	0.53	0.20
lung	0.16	0.55	0.41	0.48	0.25	0.80	0.44	0.21
tail	0.07	0.12	0.14	0.14	0.10	0.17	0.12	0.03
brain	0.00	0.00	0.01	0.01	0.00	0.02	0.01	0.00
tumor	5.46	0.95	3.17	1.06	6.30	1.62	3.09	2.11
bone	0.40	0.61	0.94	1.07	0.43	1.51	0.83	0.39
muscle	0.07	0.08	0.07	0.13	0.05	0.10	0.08	0.02
**[^198^Au]6b i.p. [MBq]**	**5.22**	**5.09**	**4.99**	**4.91**	**4.95**	**5.04**	**5.03**	
	**Mouse 49**	**Mouse 50**	**Mouse 51**	**Mouse 52**	**Mouse 53**	**Mouse 54**	**%ID/g** **n = 6**	**n = 6**
intestine small	1.82	2.44	1.17	0.92	1.32	1.31	1.50	0.50
intestine large	1.74	1.31	1.82	1.76	1.47	1.08	1.53	0.27
stomach	0.66	0.84	1.02	1.63	0.68	0.74	0.93	0.34
spleen	17.77	15.20	12.58	15.36	13.35	13.30	14.59	1.75
pancreas	2.83	3.94	3.47	3.04	5.50	4.51	3.88	0.91
liver	5.46	5.32	4.68	5.46	5.76	5.00	5.28	0.35
kidney	1.32	1.28	1.20	1.34	1.33	1.35	1.30	0.05
blood	0.03	0.03	0.02	0.04	0.03	0.06	0.03	0.01
heart	0.37	0.31	0.36	0.36	0.33	0.37	0.35	0.02
lung	0.33	0.38	0.50	0.24	0.41	0.56	0.40	0.11
tail	0.17	0.13	0.15	0.15	0.16	0.15	0.15	0.01
brain	0.01	0.01	0.00	0.00	0.01	0.01	0.01	0.00
tumor	0.47	0.17	0.17	0.27	0.22	0.23	0.25	0.10
bone	0.64	0.66	0.88	0.67	0.77	0.82	0.74	0.09
muscle	0.05	0.06	0.11	0.08	0.05	0.05	0.07	0.02

**Table A4:** Pathologic results for 36 extracted tumors. * Only positive, living cells were counted.

AuNP	HE Evaluation	CD31 Evaluation	Caspase 3 Evaluation *	α_v_β_3_ Integrin	
				% Positive Expression	Intensity
**3**	mass forming, undifferentiated (cells look atypical loss of nuclear polarity, hyperchromatic nuclei), in between stroma, and a group of atypical cells with cytoplasmic clearing (20–30%); high stromal content, grows in cord-like pattern	5–10%; scattered elongated vessels	<5%	75–80%	moderate–strong
**3**	mass forming, undifferentiated (cells look atypical loss of nuclear polarity, hyperchromatic nuclei), in between stroma, in the middle eosinophilic acellular material, and a group of atypical cells with cytoplasmic clearing (15–20%); high stromal content, grows in cord-like pattern	15–20%; scattered elongated vessels	25–30%	85–90%	moderate–strong
**3**	mass forming, undifferentiated (cells look atypical loss of nuclear polarity, hyperchromatic nuclei), in between stroma, in the middle eosinophilic acellular material, and a group of atypical cells with cytoplasmic clearing (80–90%); high stromal content, grows in cord-like pattern	10–15%; mostly in periphery	10–15%	85–90%	moderate–strong
**3**	mass forming, undifferentiated (cells look atypical loss of nuclear polarity, hyperchromatic nuclei), in between stroma, in the middle eosinophilic acellular material, and a group of atypical cells with cytoplasmic clearing (60–70%); high stromal content, grows in cord-like pattern	5–10%; mostly in periphery	10–15%	80–85%	moderate–strong
**3**	mass forming, undifferentiated (cells look atypical loss of nuclear polarity, hyperchromatic nuclei), in between stroma, in the middle eosinophilic acellular material, and a group of atypical cells with cytoplasmic clearing (80–90%); high stromal content, grows in cord-like pattern	15–20%; scattered elongated vessels	10–15%	80–85%	moderate
**3**	mass forming, undifferentiated (cells look atypical loss of nuclear polarity, hyperchromatic nuclei), in between stroma, and a group of atypical cells with cytoplasmic clearing (5%); high stromal content, grows in cord-like pattern	5–10%; scattered	5–10% (weak signal)	80–85%	moderate–strong
**3**	mass forming, undifferentiated (cells look atypical loss of nuclear polarity, hyperchromatic nuclei), in between stroma, and a group of atypical cells with cytoplasmic clearing (5%); high stromal content, grows in cord-like pattern	15–20%; scattered elongated vessels	25–30%	85–90%	moderate–strong
**3**	mass forming, undifferentiated (cells look atypical loss of nuclear polarity, hyperchromatic nuclei), in between stroma the eosinophilic acellular material; and a group of atypical cells with cytoplasmic clearing (10–15%); high stromal content, grows in cord-like pattern	10–15%; scattered elongated vessels	5–10%	>90%	moderate–strong
**3**	mass forming, undifferentiated (cells look atypical loss of nuclear polarity, hyperchromatic nuclei), in between stroma and a group of atypical cells with cytoplasmic clearing (5–10%); gland/lumen forming area (5–10%); eosinophilic acellular material; high stromal content, grows in cord-like pattern	15–20%; scattered elongated vessels	10–15%	85–90%	moderate–strong
**3**	mass forming, undifferentiated (cells look atypical loss of nuclear polarity, hyperchromatic nuclei), in between stroma and a group of atypical cells with cytoplasmic clearing (50–60%); eosinophilic acellular material; high stromal content, grows in cord-like pattern	10–15%; scattered elongated vessels	15–20%	75–80%	mild–moderate
**3**	mass forming, undifferentiated (cells look atypical loss of nuclear polarity, hyperchromatic nuclei), in between stroma and a group of atypical cells with cytoplasmic clearing (5%); gland/lumen forming area (10–15%); eosinophilic acellular material; high stromal content, grows in cord-like pattern	15–20%; scattered elongated vessels	15–20%	80–85%	moderate
**3**	mass forming, undifferentiated (cells look atypical loss of nuclear polarity, hyperchromatic nuclei), in between stroma and a group of atypical cells with cytoplasmic clearing (5–10%); the eosinophilic acellular material; high stromal content, grows in cord-like pattern	10–15%; scattered elongated vessels	10–15%	85–90%	moderate–strong
**6a**	mass forming, undifferentiated (cells look atypical loss of nuclear polarity, hyperchromatic nuclei), in between stroma and a group of atypical cells with cytoplasmic clearing (5%); gland/lumen forming area (5–10%); the eosinophilic acellular material; high stromal content, grows in cord-like pattern	15–20%; mostly in periphery	5–10%	75–80%	moderate
**6a**	mass forming, undifferentiated (cells look atypical loss of nuclear polarity, hyperchromatic nuclei), in between stroma and a group of atypical cells with cytoplasmic clearing (40–50%); the eosinophilic acellular material; high stromal content, grows in cord-like pattern	5–10%; scattered elongated vessels	10–15%	80–85%	moderate
**6a**	mass forming, undifferentiated (cells look atypical loss of nuclear polarity, hyperchromatic nuclei), in between stroma a group of atypical cells with cytoplasmic clearing (30–35%); and gland/lumen forming area (35–40%); the eosinophilic acellular material; high stromal content, grows in cord-like pattern	15–20%; scattered elongated vessels	10–15%	>90%	strong
**6a**	mass forming, undifferentiated (cells look atypical loss of nuclear polarity, hyperchromatic nuclei), in between stroma a group of atypical cells with cytoplasmic clearing (5%); and gland/lumen forming area (20–25%); the eosinophilic acellular material; high stromal content, grows in cord-like pattern	10–15%; scattered elongated vessels	10–15%	80–85%	moderate–strong
**6a**	mass forming, undifferentiated (cells look atypical loss of nuclear polarity, hyperchromatic nuclei), in between stroma and a group of atypical cells with cytoplasmic clearing (5%); gland/lumen forming area (60–65%); the eosinophilic acellular material; high stromal content, grows in cord-like pattern	15–20%; mostly in periphery	5–10%	80–85%	moderate–strong
**6a**	mass forming, undifferentiated (cells look atypical loss of nuclear polarity, hyperchromatic nuclei), in between stroma and a group of atypical cells with cytoplasmic clearing (60–70%); the eosinophilic acellular material; high stromal content, grows in cord-like pattern	10–15%; mostly in periphery	5–10%	85–90%	moderate–strong
**6a**	mass forming, undifferentiated (cells look atypical loss of nuclear polarity, hyperchromatic nuclei), in between stroma and a group of atypical cells with cytoplasmic clearing (5–10%); the eosinophilic acellular material; high stromal content, grows in cord-like pattern	5–10%; scattered vessels	<5%	85–90%	strong
**6a**	mass forming, undifferentiated (cells look atypical loss of nuclear polarity, hyperchromatic nuclei), in between stroma and a group of atypical cells with cytoplasmic clearing (40–45%); the eosinophilic acellular material; high stromal content, grows in cord-like pattern	10–15%; scattered vessels	10–15%	80–85%	moderate–strong
**6a**	mass forming, undifferentiated (cells look atypical loss of nuclear polarity, hyperchromatic nuclei), in between stroma and a group of atypical cells with cytoplasmic clearing (10–15%); gland/lumen forming area (<5%); the eosinophilic acellular material; high stromal content, grows in cord-like pattern	15–20%; mostly in periphery	20–25%	85–90%	moderate–strong
**6a**	mass forming, undifferentiated (cells look atypical loss of nuclear polarity, hyperchromatic nuclei), in between stroma and a group of atypical cells with cytoplasmic clearing (60–70%); the eosinophilic acellular material; high stromal content, grows in cord-like pattern	10–15%; scattered elongated vessels	20–25%	80–85%	mild–moderate
**6a**	mass forming, undifferentiated (cells look atypical loss of nuclear polarity, hyperchromatic nuclei), in between stroma and a group of atypical cells with cytoplasmic clearing (10–15%); the eosinophilic acellular material; high stromal content, grows in cord-like pattern	15–20%; mostly in periphery	20–25%	85–90%	moderate–strong
**6a**	mass forming, undifferentiated (cells look atypical loss of nuclear polarity, hyperchromatic nuclei), in between stroma and a group of atypical cells with cytoplasmic clearing (15–20%); gland/lumen forming area (15–20%); eosinophilic acellular material; high stromal content, grows in cord-like pattern	10–15%; mostly in periphery	10–15% (weak)	85–90%	moderate–strong
**6b**	mass forming, undifferentiated (cells look atypical loss of nuclear polarity, hyperchromatic nuclei), in between stroma and a group of atypical cells with cytoplasmic clearing (<5%); gland/lumen forming area (70–75%); eosinophilic acellular material; high stromal content, grows in cord-like pattern	15–20%; mostly in periphery	10–15%	85–90%	moderate–strong
**6b**	mass forming, undifferentiated (cells look atypical loss of nuclear polarity, hyperchromatic nuclei), in between stroma and a group of atypical cells with cytoplasmic clearing (<5%); prominent eosinophilic acellular material; high stromal content, grows in cord-like pattern	10–15%; scattered elongated vessels	15–20%	85–90%	moderate-strong
**6b**	mass forming, undifferentiated (cells look atypical loss of nuclear polarity, hyperchromatic nuclei), in between stroma, in the middle eosinophilic acellular material, and a group of atypical cells with cytoplasmic clearing (30–40%); high stromal content, grows in cord-like pattern;	10–15%; mostly in periphery	5–10%	80–85%	moderate–strong
**6b**	mass forming, undifferentiated (cells look atypical loss of nuclear polarity, hyperchromatic nuclei), in between stroma, in the middle eosinophilic acellular material, and a group of atypical cells with cytoplasmic clearing (60–65%); high stromal content, grows in cord-like pattern	10–15%; mostly in periphery	5–10%	75–80%	moderate
**6b**	mass forming, undifferentiated (cells look atypical loss of nuclear polarity, hyperchromatic nuclei), in between stroma, in the middle eosinophilic acellular material, and a group of atypical cells with cytoplasmic clearing (10–15%); high stromal content, grows in cord-like pattern	20–25%; mostly in periphery	<5%	85–90%	moderate–strong
**6b**	mass forming, undifferentiated (cells look atypical loss of nuclear polarity, hyperchromatic nuclei), in between stroma, in the middle eosinophilic acellular material, and a group of atypical cells with cytoplasmic clearing (80–90%); high stromal content, grows in cord-like pattern	5–10%; predominantly in periphery	5–10%	85–90%	moderate–strong
**6b**	mass forming, undifferentiated (cells look atypical loss of nuclear polarity, hyperchromatic nuclei), in between stroma, in the middle eosinophilic acellular material, and a group of atypical cells with cytoplasmic clearing (15–20%); high stromal content, grows in cord-like pattern	10–15%; scattered vessels	10–15%	80–85%	moderate
**6b**	mass forming, undifferentiated (cells look atypical loss of nuclear polarity, hyperchromatic nuclei), in between stroma, in the middle eosinophilic acellular material, and a group of atypical cells with cytoplasmic clearing (15–20%); high stromal content, grows in cord-like pattern	15–20%; scattered elongated vessels	10–15%	80–85%	moderate
**6b**	mass forming, undifferentiated (cells look atypical loss of nuclear polarity, hyperchromatic nuclei), in between stroma, in the middle eosinophilic acellular material, and a group of atypical cells with cytoplasmic clearing (5–10%); high stromal content, grows in cord-like pattern	10–15%; scattered vessels	10–15%	75–80%	moderate
**6b**	mass forming, undifferentiated (cells look atypical loss of nuclear polarity, hyperchromatic nuclei), in between stroma, in the middle eosinophilic acellular material, and a group of atypical cells with cytoplasmic clearing (60–70%); high stromal content, grows in cord-like pattern	5–10%; predominantly in periphery	5–10%	85–90%	moderate–strong
**6b**	mass forming, undifferentiated (cells look atypical loss of nuclear polarity, hyperchromatic nuclei), in between stroma, in the middle eosinophilic acellular material, and a group of atypical cells with cytoplasmic clearing (5%); high stromal content, grows in cord-like pattern	10–15%; scattered vessels	5–10%	80–85%	moderate–strong
**6b**	mass forming, undifferentiated (cells look atypical loss of nuclear polarity, hyperchromatic nuclei), in between stroma, in the middle eosinophilic acellular material, and a group of atypical cells with cytoplasmic clearing (5%); high stromal content, grows in cord-like pattern	15–20%; scattered vessels	5–10%	60–65%	mild–moderate
